# Prescriber variation in potentially inappropriate prescribing in older populations in Ireland

**DOI:** 10.1186/1471-2296-15-59

**Published:** 2014-04-02

**Authors:** Caitriona Cahir, Tom Fahey, Conor Teljeur, Kathleen Bennett

**Affiliations:** 1 Department of Pharmacology & Therapeutics, Trinity Centre for Health Sciences, St James's Hospital, Dublin 8, Ireland; 2 HRB Centre for Primary Care Research, Royal College of Surgeons in Ireland, Division of Population Health Science, 123 St Stephens Green, Dublin 2, Ireland; 3 Health Information and Quality Authority (HIQA), George’s Court, George’s Lane, Dublin 7, Ireland

**Keywords:** Potentially inappropriate prescribing, General practice, Prescriber variation, STOPP, Older populations

## Abstract

**Background:**

Health care policy-makers look for prescribing indicators at the population level to evaluate the performance of prescribers, improve quality and control drug costs. The aim of this research was to; (i) estimate the level of variation in potentially inappropriate prescribing (PIP) across prescribers in the national Irish older population using the STOPP criteria; (ii) estimate how reliably the criteria could distinguish between prescribers in terms of their proportion of PIP and; (iii) examine how PIP varies between prescribers and by patient and prescriber characteristics in a multilevel regression model.

**Methods:**

1,938 general practitioners (GPs) with 338,375 registered patients’ ≥70 years were extracted from the Health Service Executive Primary Care Reimbursement Service (HSE-PCRS) pharmacy claims database. HSE-PCRS prescriptions are WHO ATC coded. Demographic data for claimants’ and prescribers’ are available. Thirty STOPP indicators were applied to prescription claims in 2007. Multilevel logistic regression examined how PIP varied between prescribers and by individual patient and prescriber level variables.

**Results:**

The unadjusted variation in PIP between prescribers was considerable (median 35%, IQR 30-40%). The STOPP criteria were reliable measures of PIP (average >0.8 reliability). The multilevel regression models found that only the patient level variable, number of different repeat drug classes was strongly associated with PIP (>2 drugs *v* none; adjusted OR, 4.0; 95% CI 3.7, 4.3). After adjustment for patient level variables the proportion of PIP varied fourfold (0.5 to 2 times the expected proportion) between prescribers but the majority of this variation was not significant.

**Conclusion:**

PIP is of concern for all prescribers. Interventions aimed at enhancing appropriateness of prescribing should target patients taking multiple medications.

## Background

Clinical practice guidelines and prescribing indicators have become a common feature in many healthcare systems in an attempt to reduce unwarranted physician variation in medical care, improve quality and control drug costs [1]. In particular, improving the quality of drug prescribing in older populations is a priority due to the association between potentially inappropriate prescribing (PIP) and increases in patient morbidity, mortality, adverse drug events (ADEs), hospitalisation and healthcare costs [2,3]. A number of indicators of PIP have been developed to evaluate prescribing consisting of drugs to be avoided in older people independent of diagnosis and in the context of certain diagnoses [4-6].

There has been little research on the prevalence of PIP in primary care populations or how it varies between both patients and general practitioners (GPs) [7]. The focus to date has mainly been on variation in prescribing volumes and costs which can have low validity with prescribers [8]. Quantifying and understanding the variation in PIP is important for planning interventions and the development of guidelines and incentives to improve prescribing quality in older populations [1]. It can also provide information for performance management purposes by identifying prescribers with particularly high rates of PIP for further investigation [9].

The aim of this study was to examine the variation between GPs in the prevalence of PIP in the national Irish population aged ≥ 70 years in 2007 using the STOPP criteria. The main objectives were to: (i) estimate the level of variation in PIP across the distribution of GPs; (ii) estimate how reliably the STOPP criteria could distinguish between GPs in terms of the proportion of PIP prescribed; and (iii) examine how PIP varies between GPs and by patient and GP characteristics associated with PIP in a multilevel regression model.

## Methods

### Study population

This was a national population study of patients aged ≥ 70 years in Ireland in 2007 dispensed medication through the Primary Care Reimbursement Service of the Health Service Executive in Ireland (HSE-PCRS) pharmacy claims database. The HSE-PCRS provides free health services including medications to eligible persons in Ireland. It is means tested for those < 70 years, and free to all those ≥70 years between July 2001 and December 2008. It is estimated that over 97% of older patients nationally avail of the scheme [10]. Prescriptions are coded using the WHO ATC classification system [11]. Prescriber and patient socio-demographic information, defined daily doses (DDD), strength, quantity, method and unit of drug administration, an urban/rural classification and a measure of practice deprivation is available [12]. Patients receiving prescriptions from more than one practitioner were assigned to the practitioner who prescribed for >3 consecutive months. Where more than one practitioner prescribed over a period of >3 months, the patient was assigned to their most recent practitioner.

### Measuring PIP

Thirty STOPP criteria were applied to prescription claims data for the study population. Details of the application of the STOPP criteria to the HSE-PCRS pharmacy claims database have been described previously [13]. The thirty criteria were considered applicable to pharmacy claims data without diagnosis information on a consensus basis by an expert panel of five members in geriatric pharmacotherapy, clinical pharmacology, pharmacoepidemiology and academic general practice (Additional file 1). Prescription drugs for the treatment of certain disease conditions were identified and used as proxies for diagnosis where possible e.g. dementia (ATC, N06D), Parkinson’s disease (ATC, N04) [13] The thirty STOPP criteria were also included in a composite binary indicator defined as whether or not a patient had received any PIP indicator. The number of different repeat drug classes was measured for each patient [13,14].

### Estimating reliability

The reliability of the STOPP indicators in distinguishing between GPs was determined based on previous research and a description of this methodology is provided [7]. The reliability coefficient measures how confident one can be that the observed differences in PIP between GPs result from real differences in the quality of GP prescribing. Reliability increases as variation between GPs increases and with the number of patients per GP [7]. STOPP indicators may be reliable for GPs with a large number of patients but not for GPs with small number of patients and in order to compare GPs STOPP indicators must have adequate reliability for the majority of GPs. Reliability varies between 0 (completely unreliable) and 1 (completely reliable) with > 0.7 (70%) indicating acceptable reliability and reliabilities of 0.8-0.9 (80-90%) are preferable for clinical governance, such as paying GPs for their performance on certain quality indicators [9].

### Data analysis

The overall prevalence of PIP and the prevalence per individual STOPP criteria were calculated. The proportion of PIP prescribing (STOPP composite indicator) for each GP is also presented. Reliability was measured by estimating the intracluster correlation coefficient (ICC) for each STOPP indicator and the composite indicator in a two level random intercept logistic model with no explanatory variables (“empty model”). Reliability was calculated using the Spearman-Brown prophecy formula based on median number of patients aged ≥ 70 years per GP in 2007 [7,9]. The proportion of GPs with reliability >0.7 and >0.8 for each STOPP indicator and the composite were then calculated based on the actual number of patients in each GP practice [7].

Multilevel logistic regression investigated how the STOPP composite indicator varied between GPs and by patient and GP characteristics. Multilevel unadjusted odds ratios (OR) with 95% confidence intervals (CI) were estimated in a two level random intercept logistic model for each patient level one explanatory variable (age, gender, number of different repeat drug classes) and each GP level two explanatory variables (gender, urban/rural, deprivation). Three multivariable models were estimated; (i) Model 1, a two level random intercept logistic model with patient level one explanatory variables only; (ii) Model 2, a two level random intercept logistic model with patient level one and GP level two explanatory variables and; (iii) Model 3, a two level random slope logistic model. In Models 1 and 2, the response probability for the STOPP composite indicator was allowed to vary across GPs but the effect of each patient level one explanatory variable was assumed to be the same for each GP. Model 3, allowed both the intercept and the explanatory variable, number of different repeat drug classes, to vary randomly across GPs [15]. Likelihood ratio (LR) tests were used to compare the fit of the three models.

The between GP variance and the variance partition coefficient (VPC) were estimated for all three models [15]. The VPC partitions the variance at the patient and GP level and provides an estimate of the proportion of the total residual variance of the outcome (PIP) that is explained at the GP level and represents the heterogeneity between the GPs. The VPC was initially calculated in an “empty” model including only the random parameter (PIP). Patient level and GP level variables were then introduced into the model and the percentage of proportional change in variance was calculated, representing the percentage of variation explained by the variables in the model compared with the empty model. The median odds ratio (MOR) was also calculated which quantifies the variation between GPs by comparing two patients from two randomly chosen, different GPs [16]. A MOR equal to 1 indicates no differences between GPs in the probability of prescribing a PIP indicator. The variation between GPs after controlling for patient level variables (Model 1) was examined graphically using a funnel plot of the observed versus expected number of patients with a PIP indicator and compared to the unadjusted analysis [17].

Initial data analysis and application of the STOPP criteria to the data set was performed using SAS statistical software package version 9.1 (SAS Institute Inc. Cary, NC, USA). Multilevel logistic regression was performed in STATA Version 11.2 (StataCorp, Texas, USA) [18]. Level two residuals were checked graphically for normality, heteroskedasticity and outliers.

## Results

### Descriptive statistics

**Figure 1:**
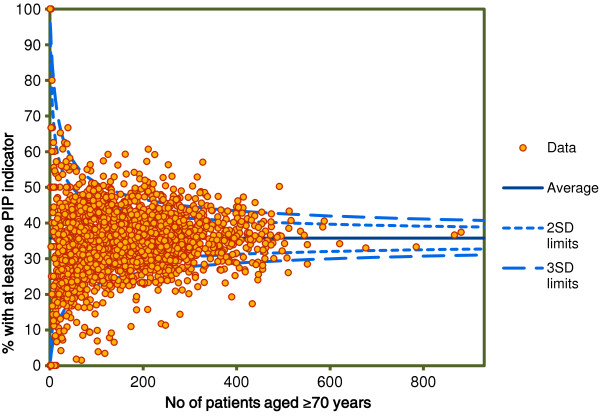
The proportion of PIP prescribing (at least one potentially inappropriate indicator) for each GP (N=1,938).

A total of 1,938 GPs and 338,725 patients aged ≥ 70 years were identified from the HSE-PCRS pharmacy claims database. The variation between GPs in the overall rate of PIP was considerable ranging from 13% at the 5^th^ percentile to 65% at the 95^th^ percentile (median 35%, IQR 29.6-40.3%). Figure 1 presents the proportion of PIP (at least one PIP indicator) for each GP. GPs outside the 3 standard deviation (SD) control limit were statistically significantly different from the average. *Common cause* variation is when the values are within the 2 SD and 3 SD lines and *special cause* variation is when the values are outside the 3 SD lines. *Common cause* variation indicates variation consistent with random chance and *special cause* variation indicates variation due to systematic influences [19]. Ninety-eight percent of GPs (N = 1,906) had at least one patient with PIP.

### Reliability of STOPP criteria

All of the thirty STOPP criteria had adequate reliability (> 0.7) based on a GP with median-sized catchment. The majority of practitioners had sufficient patient numbers aged ≥ 70 years to be reliably measured for each of the individual STOPP criteria. Between 82.6% and 99.9% of practitioners had reliability > 0.7 and between 70.1% and 97.2% had reliability > 0.8. The reliability of the composite STOPP indicator for a median sized GP was 0.80. The proportion of practitioners with sufficient patient numbers to be reliably measured was lower than the individual STOPP criteria; 76.6% had reliability > 0.7 and 60% had reliability > 0.8 (Additional file 1).

### Multilevel regression model

Table 1 shows the percentage of patients receiving at least one PIP indicator by patient and GP characteristics and the unadjusted ORs with 95% CIs estimated in a two level random intercept logistic model. PIP increased considerably with the number of different repeat drug classes; 3% of patients with no repeat prescriptions received a PIP indicator compared with 70% of those prescribed ≥10 repeat drug classes. PIP also increased with age. PIP varied less by GP level variables with a higher rate in urban based practices compared to rural.

**Table 1:** Number and percentage of patients receiving at least one potentially inappropriate indicator and multilevel unadjusted odds ratios (95% CIs)

	**Total number of patients N = 338,725**	**N (%) prescribed at least one PIP**	Multilevel unadjusted OR* (95% CIs)
**Patient level fixed effects**			
***Gender***			
Male	144,316	49,517 (34)	1
Female	194,409	71,592 (37)	1.12 (1.10, 1.13)
***Age***			
70-74 years	128,261	41,304 (32)	1
≥ 75 years	210,464	79,805 (38)	1.30 (1.28, 1.32)
***No of different repeat drug classes***			
0	28,259	950 (3)	1
1	19,533	1,278 (6)	2.01 (1.85, 2.19)
2	24,013	2,892 (12)	3.97 (3.68, 4.28)
3	28,970	5,149 (18)	6.29 (5.85, 6.75)
4	32,742	7,505 (23)	8.72 (8.13, 9.35)
5	33,538	10,078 (30)	12.64 (11.80, 13.54)
6	32,984	12,089 (37)	17.14 (16.00, 18.36)
7	29,738	12,984 (44)	22.94 (21.41, 24.57)
8	25,574	12,753 (50)	29.58 (27.59, 31.71)
9	20,908	11,755 (56)	38.12 (35.52, 40.91)
≥10	62,466	43,676 (70)	69.96 (65.40, 74.83)
**GP practice level fixed effects**^**†**^	**No of GPs N = 1,938**	**% prescribed at least one PIP**	**Multilevel unadjusted OR (95% CI)**
***Gender***^**‡**^			
Male	1,359	1,344 (99)	1
Female	557	450 (97)	0.98 (0.94, 1.01)
***Area of residence***			
Urban	1,438	1,408 (98)	1
Rural	500	470 (94)	0.89 (0.86, 0.92)
***Deprivation***	**Median**	**IQR**	
Deprivation	0.68	1.91	1.03 (1.02, 1.04)

*OR = odds ratio.

^†^GP level data was unavailable for 108 (5%) GPs with 6,906 (2%) patients.

^‡^GP gender was missing for 22 (1%) GPs with 2,578 (0.76%) patients.

Table 2 presents the adjusted ORs and 95% CIs for the three multivariate models (Models 1, 2 and 3). In the multivariate model including only patient level variables (Model 1) the association between PIP and the number of different repeat drug classes was mainly unchanged compared to the unadjusted ORs (Table 1). The associations with patient gender and age were reversed, with older patients and female patients slightly less likely to receive a PIP indicator. In the two level random intercept logistic model with no explanatory variables (empty model) the between GP variance was 9% (SE 0.004). In Model 1, after adjusting for patient level variables the residual between GP variance in receiving a PIP indicator was 6% (SE 0.003).

**Table 2:** Multilevel logistic regression adjusted odds ratios (95% CIs) for patients receiving at least one potentially inappropriate indicator

**Variable**	**Model 1 random intercept patient level variables**	**Model 2 random intercept patient and GP level variables**	**Model 3 random slope patient and GP level variables**
	OR* (95% CIs)	OR* (95% CIs)	OR* (95% CIs)
**Patient level fixed effects**			
***Gender***			
Male	1	1	1
Female	0.92 (0.90, 0.93)	0.92 (0.90, 0.93)	0.92 (0.91, 0.93) 1
***Age***			
70-74 years	1	1	10,(0.95)
≥ 75 years	0.95 (0.93, 0.97)	0.95 (0.93, 0.97)	0.95 (0.93, 0.96)
***No of different repeat drug classes***			
0	1	1	1
1	2.00 (1.83, 2.18)	2.00 (1.83, 2.18)	1.43(1.43, 1.44) †
2	3.98 (3.69, 4.30)	3.98 (3.68, 4.30)	
3	6.31 (5.87, 6.79)	6.31 (5.86, 6.78)	
4	8.81 (8.20, 9.46)	8.81 (8.20, 9.45)	
5	12.79 (11.92, 13.72)	12.78 (11.91, 13.71)	
6	17.39 (16.21, 18.65)	17.38 (16.20, 18.64)	
7	23.22 (21.65, 24.92)	23.21 (21.63, 24.90)	
8	30.15 (28.09, 32.37)	30.13 (28.07, 32.35)	
9	38.89 (36.19, 41.79)	38.86 (36.17, 41.76)	
≥10	71.77 (67.00, 76.87)	71.71 (66.95, 76.81)	
**GP level fixed effects**^**‡**^			
***Gender***^**§**^			
Male	-	1	1
Female	-	0.94 (0 .91, 0.97)	0.94 (0.91, 0.97)
***Area of residence***			
Urban	-	1	1
Rural	-	0.98 (0.95, 1.01)	0.98 (0.94, 1.01)
***Deprivation***			
Deprivation score (centred)	-	1.00 (0.99, 1.01)	1.00 (0.99, 1.01)

*OR = odds ratio.

^†^The number of different repeat drug classes was treated as a continuous variable (created 10 dummy variables and the coefficients show an approximately linear increase).

^‡^GP level data was unavailable for 108 (5%) GPs with 6,906 (2%) patients.

§GP gender was missing for 22 (1%) GPs with 2,578 (0.76%) patients.

The multivariate model including both patient and GP level variables (Model 2) had similar findings with the association between PIP and number of different repeat drug classes remaining unchanged. The association between PIP and GP area of residence (urban/rural) was no longer significant compared to the unadjusted ORs (Table 1). Adding GP level variables (Model 2) only explained an additional 0.5% of the between GP variance (Model 2 = 5.9% vs. Model 1 = 6.4%) and resulted in minimal change to the adjusted ORs. Model 3 allowed the coefficient of the number of different repeat drug classes to vary randomly across GPs. LR tests indicated that Model 3 was preferred to Models 1 and 2. Model 3 indicated that the between GP variance for PIP was a quadratic function of the number of different repeat drug classes. The lower or higher the number of repeat drug classes the more variability in PIP between GPs.

**Figure 2:**
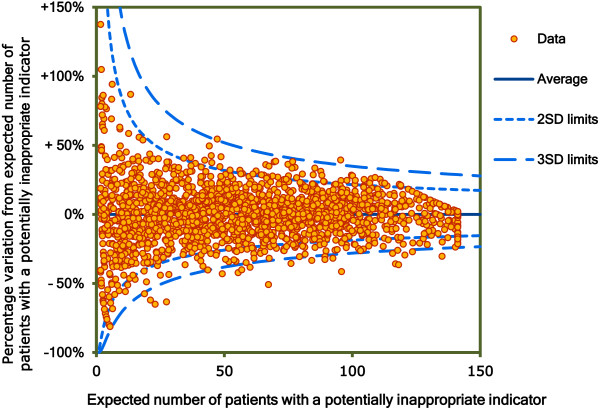
Observed versus expected number of patients with a potentially inappropriate indicator (N=1,938).

Figure 2 shows the differences between the observed numbers of patients with a PIP indicator from the expected, for each GP after adjustment for patient level explanatory variables (Model 1). GPs varied from having 50% less than the expected rates of PIP to 50% to 100% in excess but the majority of this variation was not significant (within 3 SD). GPs outside the three standard deviation control limits were statistically significantly different from the average. The VPC for the “empty” model which explains the percentage variance explained by the GP level was 2.7% and relatively small. This indicates that individual patient factors are relatively more important for PIP than GP level factors. The VPC for Model 2 was 1.76%. Thus after adjusting for the effects of patient and GP characteristics the remaining variance (1.76%) in the propensity for PIP at the GP level was attributable to unobserved patient and GP characteristics. The GP level variance was also measured by the MOR (MOR 1.26; 95% CI: 1.23 to 1.29) [16].

## Discussion

### Principal findings

There was a high prevalence of PIP in those aged ≥70 years in Ireland in 2007 and nearly all GPs had at least one patient with PIP. The most prevalent PIP drugs were PPIs at maximum therapeutic dosage for >8 weeks, followed by NSAIDs for >3 months and long-acting benzodiazepines for >1 month. The National Institute for Health and Clinical Excellence (NICE) guidelines recommend regular review of patients to assess their continuing need for PPIs and the use of step-down therapy [20]. Long-term PPI treatment has significant cost consequences [21,22]. NSAID use is associated with gastrointestinal adverse effects and hospitalisation and long-acting benzodiazepines are associated with an increased risk of falls, fractures, impaired cognition and dependence problems in older populations [23,24]. Drug duplication on the same prescription claim was also prevalent and concurrent use of NSAIDs has been shown to increase the risk of gastrointestinal toxicity [25].

The individual STOPP indicators can be used with reasonable confidence to identify GPs as having above or below average proportions of PIP (average > 0.8 reliability for 90% of GPs). Although the composite indicator had lower reliability, it is likely in practice that the individual indicators would be used to monitor the quality of prescribing. There is also evidence that the STOPP criteria have predictive validity with an association found between STOPP and ADEs in older hospitalised patients [26].

There was relatively little variation in PIP between GPs in Ireland at the GP level, the majority of the variation was at the patient level. While there was evidence of significant variation in PIP between GPs in the unadjusted analysis (Figure 1) after adjustment for patient level variables the majority of this remaining variation was no longer significant (Figure 2). This remaining variation (significant and non-significant) was not explained by adding GP level variables to the model. The characteristics of the GPs did not substantively affect the likelihood of receiving a PIP indicator at the patient level. The multilevel logistic regression model for the STOPP composite indicator found that only the number of different repeat drug classes was strongly associated with the likelihood of receiving a potentially inappropriate indicator. Other patient and GP level variables were found to be significantly associated with PIP but their odds ratios were close to one in the adjusted multilevel models. The association between the number of different repeat drug classes and the likelihood of receiving a potentially inappropriate indicator varied across GPs; the lower or higher the number of repeat drug classes the more variability in PIP between GPs.

A recent Scottish study investigated the variation in PIP between 315 practices and 139,404 patients defined as vulnerable to ADEs using 15 indicators based on explicit national prescribing safety advice (median 12.5%, IQR, 10.1%, 15.3%) [7]. Unlike the current study, the variation between practices was considerable even after adjusting for patient case mix and practice characteristics. Practices which were statistically different from average varied from having half (-50%) the expected rates of PIP prescribing to having 50%-125% in excess. The MOR was higher than the current study (1.42, 95% CI, 1.37, 1.47) [7]. The study populations and prevalence of PIP were different for the two studies which may explain the differences in variation between prescribers [7]. Both studies did however identify considerable unexplained significant and non-significant variation in PIP between prescribers and found that practice level variables did not account for this variation (additional 0.5% in both studies); only the patient level factor number of drug classes was strongly associated with PIP [7].

This study has identified that reductions in PIP will require improvement across all GPs to reduce the average rate of PIP rather than focusing on a few select GPs (outliers). The number of different repeat medications has consistently been shown to be an independent predictor for PIP in numerous studies [7,14,27-29]. The prescription of multiple medications in older adults has also been associated with an increased risk of drug interactions, adherence problems, ADEs and drug costs [27,30]. There is some evidence that interventions targeting polypharmacy in older people, using pharmaceutical care or computerised decision support, are successful in reducing medication related problems such as PIP. These and other forms of interventions that help the prescriber modify or reduce PIP in older patients should be developed and evaluated in randomised controlled trials [30].

### Strengths and limitations

This study has a number of limitations. The lack of diagnostic information in the database limited the applicability of all of the STOPP criteria and the investigation of individual patient factors and differences in drug indication. It is likely that estimates of PIP and comparisons across GPs are conservative. [31] There was a possibility of confounding by indication and patient case mix when comparing PIP rates across different GPs. However, the variable number of different repeat drug classes should account for most of the unmeasured variability in patient co-morbidities between GPs.

There was a small proportion of patients (3.5%) who were assigned to more than one GP in 2007 and these patients were assigned to the GP who prescribed their medication on a consistent basis, or their most recent GP if more than one GP prescribed their medication on a consistent basis. Therefore, a certain proportion of prescribing is unaccounted for in the analysis which may result in a more conservative estimate of PIP. In addition, the database does not include OTC items, although this is not likely to be a significant factor as the scheme provides free medical treatment and patients must pay for OTC items.

The multilevel approach used in this study controlled for confounding by including both patient and GP level predictors of PIP. In general GP variation in prescribing reflects different therapeutic approaches to health problems in older populations but the current study found minimum variation in PIP between GPs. Also none of the available patient and GP level factors could explain the remaining variation in PIP between GPs. The database had a limited number of GP and patient variables, hence limiting the ability to explain all of the remaining variance. Further multilevel research is required to investigate and understand which factors influence PIP at the different levels of health care organisation; patient, GP, and practice organisation and culture [7,32].

 Notwithstanding the limitations, this study is one of the first studies to examine how PIP varies between both patients and GPs in a national older population [7]. The application of PIP indicators to prescription databases at the patient, GP and practice level provides useful information for assessing and comparing prescribing at the population level [33].

### Policy implications

The development of PIP guidelines and their implementation is expensive and must bring value in terms of improved prescribing quality and patient outcomes. Studies on the effectiveness of clinical guidelines have been conflicting but they are effective if well constructed and implemented consistently. Guidelines also need to be closely monitored and prescribers educated to comply with them [34-36]. The introduction of regulatory prescribing guidelines were poorly followed in France because of the volume, lack of information systems and limited capacity for monitoring [37]. While in the UK, education on the use of guidelines on prescribing nutritional supplements significantly reduced total prescribing by 15% and inappropriate prescribing from 77% to 59% [38]. The use of computerised clinical decision support, academic detailing and pharmacist intervention has had some success in reducing PIP and further research on their implementation is required [39-42].

There is also evidence that guidelines are effective if accompanied by pay for performance financial incentives [43]. Performance measurements do offer an efficient mechanism to regulate health care providers, increase accountability and encourage quality improvement and care but can alienate providers and make them obstinate to change [44-46]. Not all PIP measured in prescribing databases may be inappropriate and screening tools will never be substitutes for clinical assessment and judgment. However they can be used to identify high rates of PIP and monitor and improve prescribing practices in older populations.

## Conclusion

Optimisation of drug prescribing in older patients is becoming an important public health issue worldwide and effective mechanisms and policies are needed to reduce the occurrence of PIP across all GPs and improve the quality of prescribing [31].

## Competing interests

The authors declare that they have no competing interests.

## Authors’ contributions

All authors have met the conditions of authorship credit. All authors (CC, TF, KB and CT) planned and designed the study. CC analysed the study data. KB and CT advised on the analysis. CC drafted the manuscript and all authors (CC, TF, KB, CT) critically reviewed and approved the final manuscript.

## Pre-publication history

The pre-publication history for this paper can be accessed here:

http://www.biomedcentral.com/1471-2296/15/59/prepub

## Supplementary Material

Additional file 1 Prevalence and reliability of individual STOPP criteria measures of PIP in 2007. Click here for file

## Access to the data

All authors had full access to all of the data in the study and can take responsibility for the integrity of the data and the accuracy of the data analysis.

## References

[B1] Hjerpe P Ohlsson H Lindblad U Boström K Merlo J Understanding adherence to therapeutic guidelines: a multilevel analysis of statin prescription in the skaraborg primary care database Eur J Clin Pharmacol 2011 67 4 415 23 21190018 10.1007/s00228-010-0973-4

[B2] Laroche ML Charmes JP Nouaille Y Picard N Merle L Is inappropriate medication use a major cause of adverse drug reactions in the elderly? Br J Clin Pharmacol 2007 63 2 177 86 17166186 10.1111/j.1365-2125.2006.02831.x PMC2000580

[B3] Lau DT Kasper JD Potter DEB Lyles A Bennett RG Hospitalization and death associated with potentially inappropriate medication prescriptions among elderly nursing home residents Arch Intern Med 2005 165 1 68 74 15642877 10.1001/archinte.165.1.68

[B4] Fick DM Cooper JW Wade WE Waller JL Maclean JR Beers MH Updating the beers criteria for potentially inappropriate medication use in older adults: results of a US consensus panel of experts Arch Intern Med 2003 163 22 2716 24 14662625 10.1001/archinte.163.22.2716

[B5] The American Geriatrics Society Updated beers criteria for potentially inappropriate medication use in older adults J Am Geriat Soc 2012 60 4 616 31 22376048 10.1111/j.1532-5415.2012.03923.x PMC3571677

[B6] Gallagher P Ryan C Byrne S Kennedy J O’Mahony D STOPP (screening tool of older Person’s prescriptions) and START (screening tool to alert doctors to right treatment). Consensus validation Int J Clin Pharmacol Ther 2008 46 2 72 83 18218287 10.5414/cpp46072

[B7] Guthrie B McCowan C Davey P Simpson CR Dreischulte T Barnett K High risk prescribing in primary care patients particularly vulnerable to adverse drug events: cross sectional population database analysis in Scottish general practice BMJ 2011 342 d3514 21693525 10.1136/bmj.d3514

[B8] Rasmussen HMS Søndergaard J Kampmann JP Andersen M General practitioners prefer prescribing indicators based on detailed information on individual patients: a Delphi study Eur J Clin Pharmacol 2005 61 3 237 41 15864571 10.1007/s00228-004-0870-9

[B9] Roland M Elliott M Lyratzopoulos G Barbiere J Parker RA Smith P Bower P Campbell J Reliability of patient responses in pay for performance schemes: analysis of national general practitioner patient survey data in England BMJ 2009 339 b3851 19808811 10.1136/bmj.b3851 PMC2754504

[B10] Naughton C Bennett K Feely J Prevalence of chronic disease in the elderly based on a national pharmacy claims database Age Ageing 2006 35 6 633 6 17047009 10.1093/ageing/afl106

[B11] WHO Collaborating Centre for Drug Statistics Methodology Anatomical Therapeutic Chemical (ATC) Classification Index 2010 Oslo, Norway: WHO Collaborating Centre for Drug Statistics Methodology

[B12] Teljeur C O’Dowd T Thomas S Kelly A The distribution of GPs in Ireland in relation to deprivation Health Place 2010 16 6 1077 83 20655795 10.1016/j.healthplace.2010.06.011

[B13] Cahir C Fahey T Teeling M Teljeur C Feely J Bennett K Potentially inappropriate prescribing and cost outcomes for older people: a national population study Br J Clin Pharmacol 2010 69 5 543 52 20573091 10.1111/j.1365-2125.2010.03628.x PMC2856056

[B14] Carey IM De Wilde S Harris T Victor C Richards N Hilton SR Cook DG What factors predict potentially inappropriate primary care prescribing in older people? Drugs Aging 2008 25 8 639 706 10.2165/00002512-200825080-00006 18665661

[B15] Rabe-Hesketh S Skrondal A Multilevel and Longitudinal Modeling Using Stata 2008 Texas: Stata Press

[B16] Larsen K Merlo J Appropriate assessment of neighborhood effects on individual health: integrating random and fixed effects in multilevel logistic regression Am J Epidemiol 2005 161 1 81 8 15615918 10.1093/aje/kwi017

[B17] Spiegelhalter D Funnel plots for institutional comparison Qual Safe Health Care 2002 11 4 390 1 10.1136/qhc.11.4.390-a PMC1757996 12468705

[B18] Steele F Multilevel models for binary responses 2008 Bristol: Stata Online Centre for Multilevel Modelling

[B19] Deming WE On probability as a basis for action Am Statistican 1975 29 4 146 52

[B20] NHS National Institute for Excellence (NICE) Management of dyspepsia in adults in primary care 2004 London http://guidance.nice.org.uk/CG17

[B21] Cahir C Fahey T Tilson L Teljeur C Bennett K Proton pump inhibitors: potential cost reductions by applying prescribing guidelines BMC Health Serv Res 2012 12 1 408 23163956 10.1186/1472-6963-12-408 PMC3529111

[B22] Forgacs I Loganayagam A Overprescribing proton pump inhibitors BMJ 2008 336 7634 2 3 18174564 10.1136/bmj.39406.449456.BE PMC2174763

[B23] Howard RL Avery AJ Slavenburg S Royal S Pipe G Lucassen P Pirmohamed M Which drugs cause preventable admissions to hospital? A systematic review Br J Clin Pharmacol 2007 63 2 136 47 16803468 10.1111/j.1365-2125.2006.02698.x PMC2000562

[B24] Tinetti ME Preventing falls in elderly persons N Engl J Med 2003 348 1 42 9 12510042 10.1056/NEJMcp020719

[B25] Yayla ME Bilge U Binen E Keskin A The Use of START/STOPP criteria for elderly patients in primary care Sci World J 2013 165873 4 doi:10.1155/2013/165873 10.1155/2013/165873 PMC3694555 23853529

[B26] Hamilton H Gallagher P Ryan C Byrne S O’Mahony D Potentially inappropriate medications defined by STOPP criteria and the risk of adverse drug events in older hospitalized patients Arch Intern Med 2011 171 11 1013 9 21670370 10.1001/archinternmed.2011.215

[B27] Kirk S Parker D Claridge T Esmail A Marshall M Patient safety culture in primary care: developing a theoretical framework for practical use Qual Safe Health Care 2007 16 4 313 20 10.1136/qshc.2006.018366 PMC2464946 17693682

[B28] Agostini JV Measuring drug burden: a step forward Arch Intern Med 2007 167 8 753 4 17452536 10.1001/archinte.167.8.753

[B29] Ay P Akici A Harmanc H Drug utilization and potentially inappropriate drug use in elderly residents of a community in Istanbul, Turkey Int J Clin Pharmacol Ther 2005 43 4 195 202 15966466 10.5414/cpp43000

[B30] Chang C-B Chen J-H Wen C-J Kuo H-K Lu IS Chiu L-S Wu SC Chan DC Potentially inappropriate medications in geriatric outpatients with polypharmacy: application of six sets of published explicit criteria Br J Clin Pharmacol 2011 72 3 482 9 21557760 10.1111/j.1365-2125.2011.04010.x PMC3175518

[B31] Patterson SM Hughes C Kerse N Cardwell CR Bradley MC Interventions to improve the appropriate use of polypharmacy for older people Cochrane Database Syst Rev 2012 5 CD008165 10.1002/14651858.CD008165.pub2 22592727

[B32] Spinewine A Schmader KE Barber N Hughes C Lapane KL Swine C Hanlon JT Appropriate prescribing in elderly people: how well can it be measured and optimised? Lancet 2007 370 9582 173 84 17630041 10.1016/S0140-6736(07)61091-5

[B33] Williams D Bennett K Feely J The application of prescribing indicators to a primary care prescription database in Ireland Eur J Clin Pharmacol 2005 61 2 127 33 15711833 10.1007/s00228-004-0876-3

[B34] Garfield FB Garfield JM Clinical judgement and clinical practice guidelines Int J Technol Assess 2000 16 04 1050 60 10.1017/s0266462300103113 11155827

[B35] Perleth M Jakubowski E Busse R What is ‘best practice’ in health care? State of the art and perspectives in improving the effectiveness and efficiency of the European health care systems Health Policy 2001 56 3 235 50 11399348 10.1016/s0168-8510(00)00138-x

[B36] Gundersen L The effect of clinical practice guidelines on variations in care Ann Intern Med 2000 133 4 317 10929192 10.7326/0003-4819-133-4-200008150-00102

[B37] Durieux P Chaix-Couturier C Durand-Zalesk I Ravaud P From clinical recommendations to mandatory practice Int J Technol Assess 2000 16 4 969 75 10.1017/s0266462300103046 11155845

[B38] Gall MJ Harmer JE Wanstall HJ Prescribing of oral nutritional supplements in primary care: can guidelines supported by education improve prescribing practice? Clin Nutr 2001 20 6 511 5 11883999 10.1054/clnu.2001.0479

[B39] Tamblyn R Huang A Perreault R Jacques A Roy D Hanley J McLeod P Laprise R The medical office of the 21st century (MOXXI): effectiveness of computerized decision-making support in reducing inappropriate prescribing in primary care Can Med Assoc J 2003 169 6 549 56 12975221 PMC191278

[B40] Simon SR Smith DH Feldstein AC Perrin N Yang X Zhou Y Platt R Soumerai SB Computerized prescribing alerts and group academic detailing to reduce the Use of potentially inappropriate medications in older people J Am Geriatr Soc 2006 54 6 963 8 16776793 10.1111/j.1532-5415.2006.00734.x

[B41] Zermansky AG Petty DR Raynor DK Freemantle N Vail A Lowe CJ Randomised controlled trial of clinical medication review by a pharmacist of elderly patients receiving repeat prescriptions in general practice BMJ 2001 323 7325 1340 11739221 10.1136/bmj.323.7325.1340 PMC60673

[B42] Avery AJ Rodgers S Cantrill JA Armstrong S Cresswell K Eden M Elliott RA Howard R Kendrick D Morris CJ Prescott RJ Swanwick G Franklin M Putman K Boyd M Sheikh A A pharmacist-led information technology intervention for medication errors (PINCER): a multicentre, cluster randomised, controlled trial and cost-effectiveness analysis Lancet 2012 379 9823 1310 9 22357106 10.1016/S0140-6736(11)61817-5 PMC3328846

[B43] Doran T Fullwood C Kontopantelis E Reeves D Effect of financial incentives on inequalities in the delivery of primary clinical care in England: analysis of clinical activity indicators for the quality and outcomes framework Lancet 2008 372 9640 728 36 18701159 10.1016/S0140-6736(08)61123-X

[B44] Werner RM Asch DA The unintended consequences of publicly reporting quality information JAMA 2005 293 1239 44 15755946 10.1001/jama.293.10.1239

[B45] Millenson ML Pay for performance: the best worst choice Qual Safe Health Care 2004 13 5 323 4 10.1136/qshc.2004.011668 PMC1743883 15465930

[B46] Veninga C Denig P Pont L Haaijer-Ruskamp F Comparison of indicators assessing the quality of drug prescribing for asthma Health Serv Res 2001 36 143 61 11324741 PMC1089220

