# Supplementary material for: Prescriber variation in potentially inappropriate prescribing in older populations in Ireland
Source: BMC Fam Pract. 2014 Apr 2;15:59. doi: 10.1186/1471-2296-15-59 (PMC4021047; doi:10.1186/1471-2296-15-59)
Supplement: Additional file 1 — Prevalence and reliability of individual STOPP criteria measures of PIP in 2007. [file 1471-2296-15-59-S1.docx]

Table A1: Prevalence and reliability of individual STOPP criteria measures of PIP in 2007

| **Criteria Description** | **N** | **% (95% CIs)** | **Reliability for GP with median denominator size*** | **% of practices with reliability > 0.7** | **% of practices with reliability > 0.8** |
| --- | --- | --- | --- | --- | --- |
| ***All thirty STOPP criteria***  (any PIP) | 121,109 | 35.75 (35.68, 35.83) | 0.80 | 76.57 | 60.01 |
|  |  |  |  |  |  |
| ***Cardiovascular System*** |  |  |  |  |  |
| Digoxin > 125μg/day | 1,211 | 0.36 (0.35, 0.36) | 0.96 | 96.75 | 93.96 |
| Thiazide diuretic with gout | 1,216 | 0.36 (0.35, 0.36) | 0.95 | 95.15 | 90.45 |
| Beta-blocker with COPD† | 7,924 | 2.34 (2.33, 2.34) | 0.90 | 86.53 | 77.76 |
| Beta-blocker with verapamil | 800 | 0.24 (0.23, 0.24) | 0.96 | 96.39 | 93.19 |
| Aspirin and warfarin without histamine H2 receptor antagonist  (except cimetidine) or PPI† | 3,693 | 1.09 (1.08, 1.09) | 0.93 | 93.03 | 86.07 |
| Dipyridamole as monotherapy for cardiovascular secondary prevention | 219 | 0.06 (0.06, 0.07) | 0.98 | 99.02 | 96.39 |
| Aspirin > 150mg/day | 5,711 | 1.69 (1.68, 1.69) | 0.94 | 93.45 | 87.25 |
|  |  |  |  |  |  |
| ***Central Nervous System and*** ***psychotropic drugs*** |  |  |  |  |  |
| TCA† with dementia | 609 | 0.18 (0.17, 0.18) | 0.94 | 93.45 | 87.25 |
| TCA and glaucoma | 465 | 0.14 (0.13, 0.14) | 0.95 | 94.38 | 88.85 |
| TCA and opiate or calcium channel blockers | 6,944 | 2.05 (2.04, 2.06) | 0.91 | 88.85 | 80.55 |
| Long-term (> 1 month), long-acting benzodiazepine | 17,649 | 5.21 (5.19, 5.23) | 0.92 | 91.12 | 82.97 |
| Long-term (> 1 month) neuroleptics | 5,657 | 1.67 (1.67, 1.68) | 0.94 | 93.45 | 87.25 |
| Long-term (> 1 month) neuroleptics with parkinsonism | 1,295 | 0.38 (0.37, 0.38) | 0.97 | 96.75 | 93.96 |
| Anticholinergics to treat extrapyramidal side effects of neuroleptic medications | 1,526 | 0.45 (0.44, 0.45) | 0.97 | 97.16 | 94.22 |
| Phenothiazines with epilepsy | 820 | 0.24 (0.23, 0.24) | 0.94 | 94.38 | 88.85 |
| Prolonged use (i.e. > 1 week) of first-generation antihistamines | 3,248 | 0.96 (0.95, 0.96) | 0.93 | 92.67 | 85.19 |
|  |  |  |  |  |  |
| ***Gastrointestinal System*** |  |  |  |  |  |
| Prochlorperazine or metoclopramide with parkinsonism | 726 | 0.21 (0.21, 0.22) | 0.95 | 95.15 | 90.45 |
| PPI for peptic ulcer disease at maximum therapeutic dosage for > 8 weeks‡ | 56,452 | 16.67 (16.62, 16.71) | 0.86 | 82.56 | 70.12 |
|  |  |  |  |  |  |
| ***Respiratory System*** |  |  |  |  |  |
| Theophylline with COPD | 4,008 | 1.18 (1.18, 1.19) | 0.97 | 97.16 | 94.22 |
| Nebulised ipratropium with glaucoma | 50 | 0.01 (0.01, 0.02) | NA | NA | NA |
|  |  |  |  |  |  |
| ***Musculoskeletal System*** |  |  |  |  |  |
| Long-term use of NSAID† (> 3 months) | 29,555 | 8.73 (8.70, 8.75) | 0.90 | 86.07 | 76.42 |
| Warfarin and NSAID | 2,535 | 0.75 (0.74, 0.75) | 0.91 | 88.85 | 80.55 |
|  |  |  |  |  |  |
| ***Urogenital System*** |  |  |  |  |  |
| Antimuscarinic drugs with dementia | 1,568 | 0.46 (0.45, 0.46) | 0.92 | 91.12 | 82.97 |
| Antimuscarinic drugs with chronic glaucoma (> 3 months) | 0 | NA | NA | NA | NA |
|  |  |  |  |  |  |
| ***Endocrine System*** |  |  |  |  |  |
| Glibenclamide or chlorpropamide with type 2 diabetes mellitus | 976 | 0.29 (0.28, 0.29) | 0.98 | 99.85 | 97.16 |
| ***Duplicate Drug Class Prescription***  Two concurrent opiates, NSAIDs, SSRIs†, antidepressantsloop diuretics, ACE inhibitors† | 16,201§ | 4.78 (4.78, 4.80) | 0.88 | 86.07 | 76.42 |
|  |  |  |  |  |  |

*Reliability coefficient can vary between 0 (completely unreliable) and 1 (completely reliable). Reliability greater than 0.7 indicates acceptable reliability and reliabilities of 0.8-0.9 are preferable for important evaluations such as paying practitioners for their performance on certain quality indicators.

†COPD= chronic obstructive pulmonary disease, PPI= proton pump inhibitor, TCA= tricyclic antidepressant, NSAID= non-steroidal anti-inflammatory drug, SSRI= selective serotonin reuptake inhibitor, ACE inhibitors= Angiotensin converting enzyme inhibitors.

‡ PPI at maximum therapeutic dose= 40mg/daily omeprazole, pantoprazole and esomeprazole, 30mg/daily lansoprazole and 20mg/daily rabeprazole.

§ Adjusted for those receiving more than one duplicate prescription.
